# A novel mutation in *BBS7* gene causes Bardet–Biedl syndrome in a Chinese family

**Published:** 2008-12-12

**Authors:** Zhenglin Yang, Yang Yang, Peiquan Zhao, Kechun Chen, Bin Chen, Ying Lin, Fuqiang Guo, Yigong Chen, Xiaoqi Liu, Fang Lu, Yi Shi, Dingding Zhang, Shihuang Liao, Qingyou Xia

**Affiliations:** 1 The Key Sericultural Laboratory of Agricultural Ministry; College of Biotechnology, Southwest University, Beibei, Chongqing, China; 2 Center for Human Molecular Biology & Genetics, Sichuan Academy of Medical Sciences & Sichuan Provincial People’s Hospital, Sichuan, China; 3 Department of Ophthalmology, Xihua Hospital, Shanghai Jiaotong University, Shanghai, China; 4 Linshui County Hospital, Linshu, Sichuan, China; 5 Department of Ophthalmology, Sichuan Academy of Medical Sciences & Sichuan Provincial People’s Hospital, Sichuan, China; 6 Department of Neurology, Sichuan Academy of Medical Sciences & Sichuan Provincial People’s Hospital, Sichuan, China

## Abstract

**Purpose:**

To describe the clinical features of and identify a novel mutation in Bardet–Biedl syndrome 7 gene (*BBS7*) in a Chinese family.

**Methods:**

Nineteen individuals at risk for inheriting Bardet-Biedl syndrome (BBS) in a Chinese family participated in the study. Physical examination was performed and blood was drawn for DNA extraction. Linkage analysis was conducted for all known BBS loci, and mutation screening of *BBS7* gene and *BBS12* gene was performed.

**Results:**

A Chinese family with inherited BBS was identified. After performing linkage analysis on all 13 known loci, we found the disease phenotype of a Chinese family with BBS linked to a locus where *BBS7* and *BBS12* genes locate.

**Conclusions:**

This study describes a novel mutation in *BBS7* causing BBS in a Chinese family. This is the first report that a mutation in a BBS gene causes BBS in a Chinese population. These results expand the spectrum of human disease associated with mutations of *BBS7* since the initial three mutations in *BBS7* were first identified in 2003.

## Introduction

Bardet–Biedl syndrome (BBS; OMIM 209900) is an autosomal recessive disorder characterized by pleiotropic defects including obesity, retinal dystrophy, polydactyly, hypogenitalism, learning difficulties, and renal abnormalities [1,2], as well as diseases such as diabetes mellitus, hypertension, and congenital heart disease [1,3,4]. The disorder displays genetic heterogeneity. So far, fourteen genes have been mapped and cloned: including *BBS1* (11q13), *BBS2* (16q21), *BBS3* (3p12-q13), *BBS4* (15q22.3), *BBS5* (2q31), *BBS6* (20p12), *BBS7* (4q27), *BBS8* (14q32.11), *BBS9* (7p14), *BBS10* (12q21.2), *BBS11* (9q33.1), *BBS12* (4q27), *BBS13* (17q23), and *BBS14* (12q21.3) [2,5-18]. There are still about 25%–50% of BBS families without any mutation in these genes [10,19,20]. In vitro and in vivo studies of the known BBS genes and their products have demonstrated that BBS is a disorder of defective primary ciliary and basal body function, probably affecting both transport and paracrine signals such as the planar cell polarity pathway [2,10,12,21,22]. A relatively high incidence of BBS is found in the mixed Arab populations of Kuwait and in Bedouin tribes throughout the Middle East, most likely due to the high rate of consanguinity in these populations and a founder effect. Three mutations linked to BBS have been previously described in BBS7 (T211I, K237fsX296, and H323R) [17]. However, no BBS gene mutation has been reported in Chinese populations. Here we report for the first time that a novel mutation in *BBS7* gene causes BBS in a Chinese family.

## Methods

### Study subjects

Approval from the Institutional Review Board of the Sichuan Academy of Medical Sciences and Sichuan Provincial People’s Hospital, China was obtained for this study, and informed consent was obtained from all participants. The study enrolled 19 individuals from a Chinese family who were at risk for inheriting BBS. Physical examination was performed, and blood was drawn for DNA extraction. Individuals with BBS were ascertained on the basis of criteria established elsewhere, including the cardinal features such as retinal dystrophy or other ocular abnormality, obesity, learning difficulties, polydactyly, hypogenitalism (males), renal abnormality, and other clinical features [1]. Measurements were made of height and weight. Photographs were taken of the entire body including the hands, feet, and specific dysmorphic features. Renal function was tested. The eyes were examined with retinal-function testing (color-vision testing, perimetry, and dark-adaption testing) and electroretinographic studies, photography of the fundus, and fluorescein angiography. Urogenital system examination and intelligence level evaluation were also conducted.

### Genetic linkage and mutation screening

DNA was extracted from blood samples, and genetic linkage was assessed using short tandem repeat (STR) markers using established methods [23,24]. The STR markers encompassing known BBS loci including *BBS1* (D11S987, D11S4191), *BBS2* (D16S415, D16S503), *BBS3* (D3S3619, D3S1752), *BBS4* (D15S131, D15S205), *BBS5* (D2S2330, D2S335), *BBS6* (D20S115, D20S186), *BBS7*/*BBS12* (D4S402, D4S427, GATA30B11), *BBS8* (D14S68, D14280), *BBS9* (D7S516, D7S484), *BBS10* (D12S376, D12S326), *BBS11* (D9S1811, GGAT11B01), *BBS13* (D17S787, D17S944), and *BBS14* (D12S326, D12S351) were genotyped and analyzed. *BBS7* and *BBS12* mutation screening was performed by direct sequencing of PCR-amplified DNA fragments for all exons using established methods [24]. Amplified products were purified using the QIAquik Gel Extraction Kit (Qiagen, Valencia, CA) and sequenced with forward and reverse primers by the BigDye® Terminator v3.1 Cycle Sequencing Kit (ABI Applied Biosystems, Foster City, CA) according to the manufacturers’ instructions.

## Results

### Clinical findings

**Figure 1:**
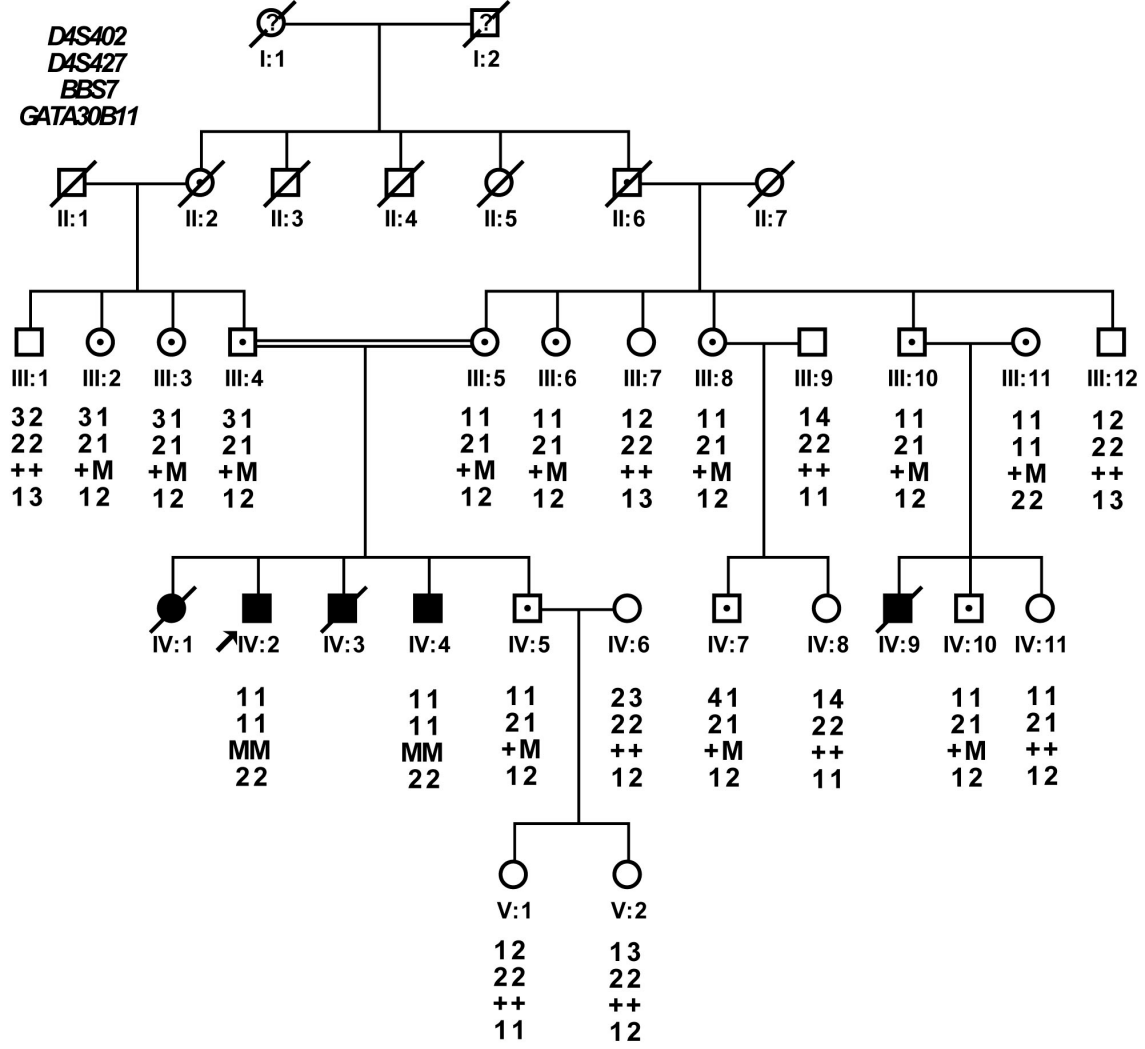
Kindred structure and segregation of the STR markers in the family. Genotyping results for short tandem repeat (STR) markers, D4S402, D4S427, GATA30B11, which tightly encompassed *BBS7*, are shown below each individual. The disease alleles of the STR markers and the mutation of *BBS7* are completely co-segregated with the phenotype of family. Affected individuals are identified by solid squares (males) or solid circles (females). Normal individuals are identified by open symbols; carry individuals are identified by open symbols with a dot inside; deceased individuals are indicated by a slash(/). Arrow indicates the proband of the family. M, mutant allele of *BBS7*; +, normal allele of *BBS7*.

**Figure 2:**
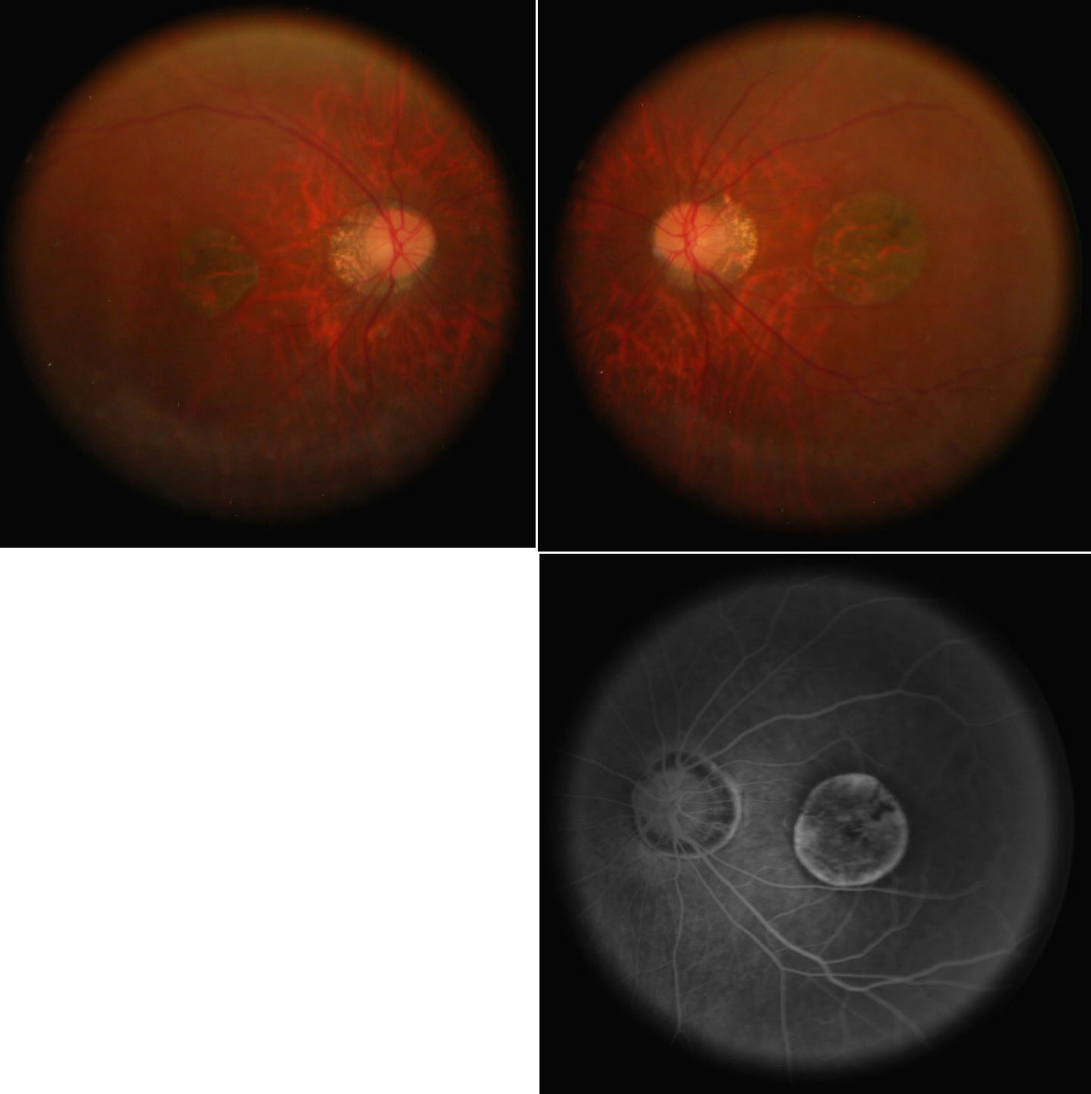
The retinal changes of the affected patients of the family. Retinal dystrophy is one of main clinical features of Bardet–Biedl syndrome (BBS). Fundus pictures (top left: OD; top right: OS) and an angiography picture (bottom right: OS) of IV: 2 of the family showed atypical retinal dystrophy with attenuated vessels, choroidal sclerosis-type change, severe optic disc pallor, and severe macular dystrophy. The fundus changes of the patient are different from the typical retinal dystrophy of retinitis pigmentosa (RP) in BBS.

A Chinese consanguineous BBS family was identified in southwest China. Of the 19 members at risk for inheriting BBS in this family, five were diagnosed with BBS (Figure 1). Two affected patients died of severe fever when they were five years of age, and one affected patient died from an accident. The two living affected patients displayed gross obesity in relation to the average height of adult Chinese men. In addition, these patients exhibited the following features: atypical retinal dystrophy with attenuated vessels, choroidal sclerosis-type change, severe optic disc pallor and severe macular dystrophy (Figure 2), obviously constricted visual fields, severe abnormalities of color vision, reduced rod and cone electroretinograph (ERG) response, refractive errors of −10 (IV:2) and −8.0 (IV:4) diopters, polydactyly of hands and feet, hypogenitalism (small testes and genitalia), learning difficulties, and renal abnormalities. The detailed clinical information for these two affected patients is listed in Table 1. Based on their symptoms, the affected patients were given a diagnosis of BBS [1,2].

**Table 1:** Clinical features of the two affected patients.

**Phenotype**	**IV:2**	**IV:4**
Gender	Male	Male
Current age (Y)	37	32
Retinal dystrophy	Yes	Yes
Ptosis	Yes	Yes
Strabismus	Yes	Yes
Obesity (BMI)	Yes (25.6)	Yes (25.2)
Developmental delay	Severe	Severe
Hypogenitalism	Yes	Yes
Learning/comprehension	Delayed	Delayed
Speech development	Delayed	Delayed
Memory	Poor	Poor
Motor skills	Delayed	Delayed
Hypotonia	Yes	Yes
Dental architecture	Normal	Normal
Tongue morphology	Enlarged	Enlarged
Voice	Raspy	Raspy
Behavior	Labile	Labile
Polydactyly	Yes	Yes
Renal abnormality	Yes	Yes
Eye movement	Restrict	Restrict
Diabetes	No	No

The two affected patients showed cardinal features of Bardet–Biedl syndrome (BBS) such as retinal dystrophy or other ocular abnormality, obesity, learning difficulties, polydactyly, hypogenitalism, renal abnormality, and other clinical features of BBS.

### Genotyping and sequencing analysis

**Figure 3:**
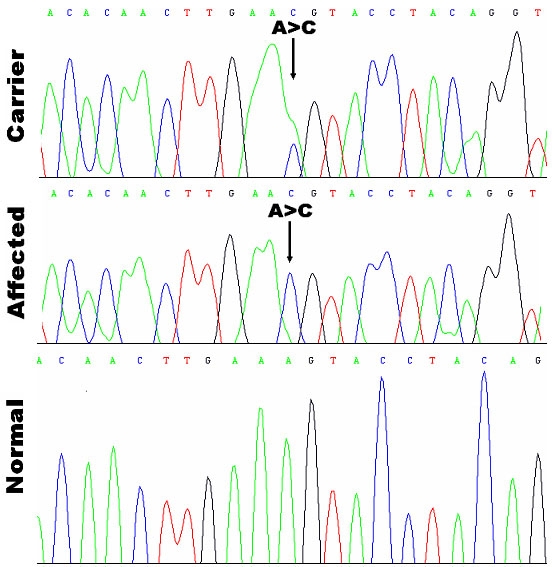
Automated sequencing traces of the patients in the family. The affected patient had an A>C transition in exon 15 of *BBS7* in the family. The carrier showed 1666 A>C heterozygous mutation (S556R), and the affected patient showed homozygous 1666 A>C mutation (S556R) compared with the normal individual in the family.

The genotyping results showed that the *BBS7/BBS12* encompassed STR markers, D4S402, D4S427, and GATA30B11, completely cosegregated with the phenotype in the family (Figure 1). But no linkage was found to any other known BBS loci. No mutation was found in *BBS12*. Mutation screening in the 19 exons of *BBS7* found a 1666 A>G mutation in exon 15, which resulted in an amino acid substitution from serine to arginine at position 556 (Figure 3). This mutation was absent in 100 normal matched controls.

## Discussion

It is clear that BBS is a pleiotropic, autosomal recessive disorder. The cardinal phenotype in this family was identical to most cases described previously [1,2] except that the ocular phenotype of this family showed macular dystrophy instead of a typical retinitis pigmentosa in BBS, further indicating the variant retinal dystrophy in different BBS families. The ocular manifestations of BBS included an early and usually severe rod-cone dystrophy that is detected by ERG and causes central and peripheral visual loss by the second to third decades of life. More than 70% of the affected individuals are legally blind by this time. High myopia associated with choroidal sclerosis-type changes in our family further confirmed the association of a myopia refractive error trend with BBS [19,25].

To date, 13 loci and 14 genes have been mapped and identified since the first BBS gene, *MKKS,* was identified in 2000 [2,18,25]. *CCDC28B* modifies the expression of BBS phenotypes in the patients who have mutations in other genes [2]. Mutations in *BBS1* constitute the most mutations in the known BBS families (about 23%). Mutations in *BBS10*, *BBS2*, and *BBS12* account for 10%, 8%, and 5% of all mutations, respectively. Other known BBS genes are rarely mutated in the known BBS families [10,19]. The mutations in these 14 genes probably affect both transport and paracrine signals, such as the planar cell polarity pathway, and cause the pleiotropic defects in affected BBS patients [2,10,12,16,18,20-22,26].

In 2003, Badano, et al. [17] performed phylogenetic and genomic studies in which they used the human and zebrafish BBS2 peptide sequences to search dbEST and the translation of the draft human genome. They identified a novel gene, *BBS2L1*. *BBS2L1* mutations cause BBS, defining a novel gene for this syndrome, *BBS7*. *BBS1*, *BBS2*, and *BBS7* share a distinct substructure, and they may belong to a distinct subfamily of proteins [2,17]. Two transcript variants encoding distinct isoforms have been identified for this gene. The length of isoform 1 is 43 amino acids longer than that of isoform 2. Isoform 2 is ubiquitously expressed, whereas isoform 1 is expressed in retina, lung, liver, testis, ovary, prostate, small intestine, liver, brain, heart, and pancreas. *BBS7* may play a role in eye, limb, cardiac, and reproductive system development. Blacque et al. [21] showed that mutations in the *Caenorhabditis elegans bbs-7* and *bbs-8* genes cause structural and functional defects in cilia. They further demonstrated that *BBS7* and *BBS8* are required for the normal localization and motility of the intraflagellar transport (IFT) proteins so the BBS7 protein plays an important role in the assembly as well as function of IFT particle components. These findings indicate that the cardinal and secondary symptoms of BBS may result from cilia [21]. How the mutation in our family caused BBS is not understood as this mutation is located closer to the C-terminal of *BBS7* compared with the three mutations reported previously. The initially identified three mutations are located in a predicted β-propeller region of *BBS7* that is conserved between *BBS2* and *BBS7*. These findings may indicate that the β-propeller region of *BBS7* and the region involving the mutation identified in this study play a critical role in the IFT system. The mutations in these regions probably disturb the localization and motility of the IFT proteins [17,21].

BBS is a rare development disorder, and 14 genes that cause BBS have been identified. However, because of the significant clinical and genetic heterogeneity of BBS, there are still unknown genes involving at least 25%–50% of BBS families [10,15,16,27]. Studies done in more families, especially from different ethnic populations, will be useful to help identify other BBS disease-causing genes and mutations and to better understand the disease mechanism of BBS. Because no BBS mutation has been identified in a large Chinese population, the novel *BBS7* mutation reported here will expand the insight into our understanding of BBS.
